# Continuous glucose monitoring use is associated with higher physical activity in adults with type 1 diabetes: a nationwide cross-sectional study

**DOI:** 10.1186/s13098-026-02238-5

**Published:** 2026-07-10

**Authors:** Jurga Šuminienė, Rimantas Stukas, Virginija Gaigalaite, Natalja Istomina

**Affiliations:** 1 Department of Public Health, Institute of Health Sciences, Faculty of Medicine, Vilnius University, M. K. Čiurlionio g. 21 Vilnius, Vilnius, LT-03101 Lithuania; 2 Institute of Clinical Medicine, Faculty of Medicine, Vilnius University, Vilnius, Lithuania; 3 Institute of Health Sciences, Faculty of Medicine, Vilnius University, Vilnius, Lithuania

**Keywords:** Continuous glucose monitoring, Diabetes distress, Hypoglycemia confidence, Moderate-to-vigorous physical activity, Physical activity, Type 1 diabetes

## Abstract

**Background:**

Physical activity is a key component of type 1 diabetes (T1D) management, yet many adults do not achieve recommended activity levels. Continuous glucose monitoring (CGM) may reduce glycemic uncertainty during exercise; however, its association with real-world physical activity remains unclear.

**Objective:**

To examine whether CGM use is independently associated with physical activity and to identify psychological factors related to perceived exercise facilitation among CGM users.

**Methods:**

In this nationwide cross-sectional study, 368 adults with self-reported T1D in Lithuania (197 CGM users and 171 non-users) completed the International Physical Activity Questionnaire–Short Form (IPAQ-SF). Multivariable linear and logistic regression models adjusted for demographic and clinical covariates were used to evaluate associations between CGM use and physical activity outcomes. Among CGM users, logistic regression was applied to identify factors associated with perceived facilitation of physical activity.

**Results:**

CGM users reported higher total physical activity (median 427.5 vs. 270 min/week, *p* = 0.001) and were more likely to engage in any moderate-to-vigorous physical activity (adjusted OR 4.4, 95% CI 2.2–8.0, *p* = 0.001) and to achieve ≥ 600 MET-min/week (adjusted OR 2.2, 95% CI 1.2–4.5, *p* = 0.035). Associations remained significant after adjustment for acute and chronic diabetes-related complications. Among CGM users, trust in CGM accuracy during exercise was strongly associated with perceived facilitation (adjusted OR 9.2, 95% CI 2.2–23.0, *p* = 0.008), whereas higher emotional diabetes-related distress was inversely associated (adjusted OR 0.65, 95% CI 0.44–0.90, *p* = 0.04).

**Conclusions:**

CGM use was independently associated with higher physical activity levels in adults with T1D. Psychological factors, particularly technology-related trust and emotional distress, may contribute to differences in perceived exercise facilitation among CGM users. These findings should be interpreted as associative rather than causal.

**Supplementary Information:**

The online version contains supplementary material available at https://doi.org/10.1186/s13098-026-02238-5.

## Introduction

Regular physical activity (PA) is a central component of type 1 diabetes (T1D) self-management and is associated with improved cardiometabolic outcomes, reduced cardiovascular risk, and better psychological well-being [1–3]. Current international guidelines recommend that adults accumulate at least 150 min per week of moderate-to-vigorous physical activity (MVPA) [1, 2]. Nevertheless, many adults with T1D fail to meet these targets, suggesting persistent barriers to sustained engagement in exercise.

Fear of hypoglycaemia is one of the most frequently reported barriers to physical activity in T1D [4–6]. Glycemic responses to exercise are highly variable and influenced by pre-exercise glucose levels, circulating insulin, exercise intensity, and duration [7, 8]. Aerobic activity, in particular, is associated with glucose declines and increased hypoglycaemia risk. Despite advances in diabetes technologies, perceived exercise-related risks remain common, underscoring the importance of understanding both physiological and behavioral determinants of activity participation.

Continuous glucose monitoring (CGM) provides real-time glucose data, trend arrows, and predictive alerts that may reduce uncertainty during exercise. Randomized controlled trials have demonstrated that CGM improves glycemic control and reduces hypoglycaemia exposure compared with conventional monitoring strategies [9–11]. However, evidence linking CGM use to higher real-world physical activity levels remains inconsistent. Systematic reviews and secondary analyses of clinical trials generally report limited changes in overall activity volume following CGM initiation [12], whereas observational studies suggest that CGM users may engage in higher levels of vigorous activity in certain populations [13, 14]. These discrepancies suggest that technological efficacy does not automatically translate into behavioral change.

Psychological factors may partly explain these mixed findings. Diabetes-related distress is common in T1D and has been associated with impaired self-management behaviors [15]. Hypoglycaemia-related confidence may influence willingness to engage in exercise despite glycemic variability [16], while broader psychological well-being may also shape health behaviors [17]. Thus, both technology-related and emotional factors may contribute to exercise participation in the context of CGM use.

In Lithuania, national reimbursement for CGM for individuals with T1D was introduced in 2022, substantially expanding access to real-time glucose monitoring. This policy change provides a natural real-world setting to examine associations between CGM use and physical activity behavior at the population level.

Therefore, this study aimed to [1] compare physical activity and sedentary behavior between CGM users and non-CGM users in a national adult T1D population and [2] identify psychological and technology-related factors associated with perceived facilitation of physical activity among CGM users.

## Materials and methods

### Study design and participants

A nationwide cross-sectional online survey was conducted between December 2023 and May 2024 among adults (≥ 18 years) with self-reported type 1 diabetes (T1D) residing in Lithuania. The survey was disseminated through national patient organizations and social media platforms. Participation was voluntary and anonymous, and electronic informed consent was obtained prior to survey initiation. The study was conducted in accordance with the Declaration of Helsinki and approved by the Vilnius University Faculty of Medicine Ethics Committee (No. 150000-KT-269).

Eligible participants were adults (≥ 18 years) with self-reported T1D who completed the questionnaire independently. Of 512 initiated surveys, 368 were completed (completion rate 71.8%) and included in the final analysis.

Although time in range (TIR) is a clinically relevant glycemic metric, these data were available for only 250 of 368 participants and were therefore not included in the main analyses because of substantial missingness.

### Glucose monitoring modality

Participants were categorized as continuous glucose monitoring (CGM) users or non-CGM users (self-monitoring of blood glucose [SMBG] or flash glucose monitoring [FGM]). Because FGM is not reimbursed in Lithuania and represented a small proportion of the sample, and because preliminary analyses showed no meaningful differences in physical activity outcomes between SMBG and FGM users, these groups were combined. Among the 171 participants classified as non-CGM users, 27 (15.8%) used FGM and 144 (84.2%) used SMBG. Given the small number of FGM users, separate analyses were not considered statistically meaningful.

Duration of CGM use was recorded among CGM users. Insulin pump therapy was described but excluded from multivariable models due to strong collinearity with CGM use.

### Covariates

Collected variables included age, sex, education (secondary/lower; college; university), residence (city/district/rural), employment (employed/student vs. other), living status (living alone vs. not), diabetes duration (years), HbA1c (%), smoking status, body mass index (BMI), and diabetes-related complications (acute and chronic). HbA1c values were self-reported by participants and were not independently verified through medical records. Covariates included in the primary models were selected a priori based on clinical relevance and previous literature regarding determinants of physical activity and CGM use in adults with T1D.

For regression analyses, education was modeled as an ordinal three-level variable, residence was dichotomized (city vs. non-city), and living status was coded as living alone (yes/no). Smoking, BMI ≥ 30 kg/m², prolonged sitting (≥ 8 h/day), and diabetes-related complications were evaluated in sensitivity analyses.

### Physical activity assessment

Physical activity was assessed using the International Physical Activity Questionnaire–Short Form (IPAQ-SF) [18]. Weekly minutes were calculated as minutes/day × days/week and truncated at 180 min/day per domain according to IPAQ scoring guidelines.

Total PA was defined as moderate + vigorous + walking; moderate-to-vigorous physical activity (MVPA) as moderate + vigorous. MET-minutes/week were calculated using standard IPAQ conversion factors. Daily sitting time was recorded as hours/day.

### Psychosocial measures

Emotional diabetes-related distress was assessed using the emotional burden subscale of the Diabetes Distress Scale (DDS-17) [15]. Hypoglycemia-related confidence was measured using the Hypoglycemia Confidence Scale [16]. Psychological well-being was assessed using the WHO-5 Well-Being Index [17].

Perceived facilitation of physical activity was assessed among CGM users using a study-specific item asking whether physical activity became easier after initiating CGM use. Responses were dichotomized as yes [1] versus no or unsure (0).

Trust in CGM accuracy during physical activity was assessed using a study-specific statement evaluating whether CGM reliably detects glucose fluctuations before, during, and after physical activity and thereby supports participation in physical activity. Responses were dichotomized as agree [1] versus disagree or unsure (0).

Both items were developed specifically for the present survey and have not undergone formal psychometric validation.

### Outcomes

Primary outcomes were:

1. Total weekly PA (minutes/week).
2. Engagement in any MVPA (> 0 min/week).

Secondary outcomes included achievement of ≥ 600 min/week of total physical activity. In sensitivity analyses, achievement of ≥ 600 MET-min/week according to IPAQ criteria was additionally evaluated.

### Statistical analysis

Continuous variables are presented as median (interquartile range [IQR]) and categorical variables as counts and percentages. Between-group comparisons were performed using the Mann–Whitney U test and chi-square test, as appropriate.

Because total physical activity in minutes/week was highly right-skewed, it was modeled as a log-transformed continuous outcome. Because some participants reported zero total physical activity, a constant was added prior to transformation and ln(total PA + 1) was used. The standardized IPAQ threshold of ≥ 600 MET-min/week was examined separately in sensitivity logistic regression analyses.

Four multivariable models were constructed:

1. Linear regression with ln(total PA + 1) as the dependent variable;
2. Logistic regression for engagement in any MVPA (> 0 min/week);
3. Logistic regression for achieving ≥ 600 MET-min/week (sensitivity analysis); and.
4. Logistic regression among CGM users to identify factors associated with perceived facilitation of physical activity.

Sensitivity analyses including insulin pump therapy and stratified analyses by pump use were considered. However, because only one participant in the non-CGM group used insulin pump therapy and because of strong collinearity between pump therapy and CGM use, these analyses were not considered statistically informative.

All primary models were adjusted a priori for age, sex, education, residence, employment, living status, diabetes duration, and HbA1c. Sensitivity models additionally included smoking status, BMI ≥ 30 kg/m², sitting time, and diabetes-related complications.

Model calibration for logistic regression was assessed using the Hosmer–Lemeshow test. Multicollinearity was evaluated using variance inflation factors (VIF < 2). Linearity in the logit was assessed for continuous predictors.

Given the limited number of outcome events in the facilitation model, a parsimonious model specification was applied to reduce the risk of overfitting. The parsimonious model included age, sex, HbA1c, emotional diabetes-related distress, and trust in CGM accuracy during physical activity.

In addition, an exploratory alternative model was constructed among CGM users including psychosocial variables (total diabetes distress, hypoglycemia-related confidence, and WHO-5 well-being) to assess the robustness of associations with perceived facilitation of physical activity.

A complete-case analysis was performed. Statistical analyses were conducted using SPSS version 24 (IBM Corp., Armonk, NY, USA). A two-sided p-value < 0.05 was considered statistically significant.

## Results

### Participant characteristics

A total of 368 adults with T1D were included; 197 (53.5%) were CGM users and 171 (46.5%) were non-CGM users (SMBG/FGM). The proportion of women did not differ between groups (58.9% vs. 58.5%, *p* = 0.90). CGM users were younger (38 [IQR 27–49] vs. 44 [33–60] years, *p* < 0.001) and more frequently had a university degree (62.4% vs. 43.3%, *p* = 0.001). HbA1c tended to be lower among CGM users (7.2% vs. 7.8%, *p* = 0.054).

No significant between-group differences were observed for residence, employment, living status (living alone: 13.2% vs. 17.0%), smoking status, or diabetes duration (all *p* > 0.05). Insulin pump use was more common among CGM users (21.8% vs. 0.6%, *p* = 0.001). Median CGM duration was 24 (IQR 12–36) months (Table 1).

**Table 1:** Participant characteristics by CGM use (*n* = 368)

Characteristic	Total sample (*n* = 368)	CGM users (*n* = 197)	Non-CGM users (*n* = 171)	*p*-value
Female sex, n (%)	216 (58.7)	116 (58.9)	100 (58.5)	0.90
Age, years, median (IQR)	40 (30–51)	38 (27–49)	44 (33–60)	< 0.001
Living alone, n (%)	55 (14.9)	26 (13.2)	29 (17.0)	0.30
Education (secondary/lower; college; university), n (%)	107/64/197 (29.1/17.4/53.5)	42/32/123 (21.3/16.2/62.4)	65/32/74 (38.1/18.7/43.3)	0.001
Residence (city/district/rural), n (%)	310/18/40 (84.2/4.9/10.9)	159/14/24 (80.7/7.1/12.2)	151/4/16 (88.3/2.3/9.4)	0.06
Employment (employed/student/other), n (%)	273/40/55 (74.2/10.9/14.9)	148/23/26 (75.1/11.7/13.2)	125/17/29 (73.1/9.9/17.0)	0.56
Diabetes duration, years, median (IQR)	13 (7–23)	13 (6–25)	14 (7–23)	0.80
Smoking, n (%)	78 (21.2)	42 (21.3)	36 (21.1)	0.90
HbA1c, median (IQR)	7.2 (6.8–8.0)	7.2 (6.8–8.0)	7.8 (6.7–8.4)	0.054
Insulin pump use, n (%)	44 (12.0)	43 (21.8)	1 (0.6)	0.001
CGM duration*, months, median (IQR)	—	24 (12–36)	—	—

Non-CGM includes SMBG and FGM users. Continuous variables were compared using the Mann–Whitney U test and categorical variables were compared using the chi-square test. *Reported only among CGM users. Insulin pump use was described but not included in multivariable models due to strong collinearity with CGM use

### Physical activity and sedentary behavior

CGM users reported higher activity levels across all domains (Table 2). Weekly moderate (135 vs. 78.7 min, *p* = 0.001), vigorous (33.7 vs. 0 min, *p* = 0.019), and walking activity (270 vs. 112.5 min, *p* = 0.001) were greater among CGM users. Accordingly, total PA (427.5 vs. 270 min/week, *p* = 0.001) and MVPA (157.5 vs. 78.8 min/week, *p* = 0.001) were significantly higher.

**Table 2:** Physical activity and sedentary behavior by CGM use

Variable	Total sample (*n* = 368)	CGM users (*n* = 197)	Non-CGM users (*n* = 171)	*p*-value
Moderate activity, min/week, median (IQR)	112.5 (0–281.25)	135 (78.5–262.5)	78.7 (0–262.5)	0.001
Vigorous activity, min/week, median (IQR)	0 (0–67.5)	33.7 (0–67.5)	0 (0–33.7)	0.019
Walking, min/week, median (IQR)	262.5 (67.5–450)	270 (112.5–450)	112.5 (67.5–270)	0.001
Total PA, min/week, median (IQR)	371.25 (157.5–637.5)	427.5 (292.5–682.5)	270 (92.8–495)	0.001
MVPA, min/week, median (IQR)	112.5 (0–281.25)	157.5 (78.75–292.5)	78.8 (0–276.5)	0.001
MVPA ≥ 150 min/week, n (%)	162 (44.0)	99 (50.3)	63 (36.8)	0.002
Total PA ≥ 600 min/week, n (%)	102 (27.7)	66 (33.5)	36 (21.1)	0.008
Sitting time, h/day, median (IQR)	5.5 (3.5–7.5)	5.5 (2–9.5)	5.5 (3.5–7.5)	0.10
High sedentary time (≥ 8 h/day), n (%)	86 (23.4)	53 (26.9)	33 (19.3)	0.09

Weekly minutes were calculated as minutes/day × days/week. Total PA = moderate + vigorous + walking; MVPA = moderate + vigorous. The IPAQ-SF scoring protocol was applied; each activity domain was truncated at 180 min/day. Continuous variables were compared using the Mann–Whitney U test and categorical variables were compared using the chi-square test

**Table 3:** Multivariable regression analyses of physical activity outcomes. Linear regression: ln(total PA minutes/week + 1)

Variable	B	SE	Standardized β	*p*-value
CGM use (yes vs. no)	0.53	0.22	0.16	0.018
Age (per year)	−0.02	0.009	−0.22	0.006
Female sex	0.38	0.22	0.11	0.09
Education (3 levels)*	−0.53	0.22	−0.14	0.019
Employment (employed/student vs. other)	0.009	0.29	0.002	0.96
Residence (city vs. non-city)	−0.50	0.29	−0.11	0.08
Living alone (yes vs. no)	−0.10	0.25	−0.03	0.69
HbA1c (%)	−0.03	0.036	−0.06	0.36
Diabetes duration (per year)	−0.001	0.012	−0.01	0.80

Model fit: R^2^ = 0.15; Adjusted R^2^ = 0.11. VIF < 2 for all variables. Education was entered as an ordinal 3-level variable

Overall, 44.0% achieved ≥ 150 min/week of MVPA, with a higher prevalence among CGM users (50.3% vs. 36.8%, *p* = 0.002). Similarly, 27.7% of participants accumulated ≥ 600 min/week of total physical activity (descriptive threshold) (33.5% vs. 21.1%, *p* = 0.008). This descriptive threshold based on total activity minutes is distinct from the standardized IPAQ classification of ≥ 600 MET-min/week used in sensitivity regression analyses.

Using the standardized IPAQ classification, CGM users were less likely than non-CGM users to fall below 600 MET-min/week (12.7% vs. 42.5%; overall distribution *p* < 0.001).

Daily sitting time and the prevalence of high sedentary behavior (≥ 8 h/day) did not differ significantly between groups.

### Multivariable associations with physical activity

In adjusted linear regression, CGM use was independently associated with higher ln (total PA + 1) (B = 0.53, SE = 0.22, *p* = 0.018) (Table 3).

Older age and higher education level were independently and negatively associated with total PA (B = − 0.02 per year, *p* = 0.006; B = − 0.53, *p* = 0.019, respectively).

In adjusted logistic regression, CGM use was strongly associated with engagement in any MVPA (> 0 min/week) (adjusted OR 4.4, 95% CI 2.2–8.0, *p* = 0.001) (Table 4).

Participants living alone had higher odds of engaging in any MVPA (adjusted OR 3.0, 95% CI 1.2–6.0, *p* = 0.021).

### Diabetes-related complications

Non-CGM users had a higher prevalence of several diabetes-related complications, including any chronic complication (56.1% vs. 47.4%,*p* = 0.034), retinopathy (39.2% vs. 21.8%,*p* = 0.001), nephropathy (31.9% vs. 8.1%,*p* = 0.001), and severe hypoglycemia in the prior six months (27.5% vs. 5.6%,*p* < 0.001) (Supplementary Table S1). However, additional adjustment for both acute and chronic complications did not materially change the observed associations between CGM use and physical activity outcomes.

### Perceived facilitation among CGM users

Among CGM users, greater trust in CGM accuracy during physical activity (adjusted OR 9.2, 95% CI 2.2–23.0, *p* = 0.008) and lower emotional diabetes-related distress (adjusted OR 0.65, 95% CI 0.44–0.90, *p* = 0.04) were independently associated with reporting that physical activity became easier after CGM initiation (Table 5). Age, sex, and HbA1c were not independently associated in the parsimonious model.

**Table 4:** Logistic regression: Engagement in any MVPA (> 0 min/week)

Variable	Adjusted OR	95% CI	*p*-value
CGM use (yes vs. no)	4.4	2.2–8.0	0.001
Age (per year)	0.97	0.95–1.00	0.059
Female sex	0.9	0.48–1.6	0.80
Education (3 levels)*	1.2	0.87–1.8	0.30
Employment (employed/student vs. other)	0.75	0.32–1.6	0.50
Residence (city vs. non-city)	1.4	0.7–3.0	0.30
Living alone (yes vs. no)	3.0	1.2–6.0	0.021
HbA1c (%)	0.98	0.80–1.01	0.40
Diabetes duration (per year)	0.98	0.95–1.01	0.27

*Education categorized as secondary or lower; college/non-university higher; university degree. Residence dichotomized as city vs. non-city (district town + rural). All models adjusted a priori for age, sex, education, residence, employment, living status (living alone vs. not), diabetes duration, and HbA1c

**Table 5:** Factors associated with perceived facilitation of physical activity among CGM users (parsimonious model)

Variable	Adjusted OR	95% CI	*p*-value
Emotional diabetes-related distress (per 1-point increase)	0.65	0.44–0.90	0.04
Trust in CGM accuracy during physical activity (yes vs. no)	9.2	2.2–23.0	0.008
Age (per year)	1.01	0.96–1.06	0.65
Female sex	0.8	0.3–2.1	0.67
HbA1c (%)	0.97	0.80–1.40	0.90

Multivariable logistic regression performed among CGM users with a definitive yes/no response regarding perceived facilitation. The model was specified parsimoniously to reduce overfitting, considering the events-per-variable (EPV) ratio

In an alternative psychosocial model, trust in CGM accuracy remained independently associated, whereas total diabetes distress, hypoglycemia-related confidence, and WHO-5 well-being were not (Supplementary Table S4).

Only 25 of 197 CGM users (12.7%) reported increased physical activity after CGM initiation. Exploratory univariable analyses suggested an association between longer diabetes duration and increased activity; however, no consistent psychosocial predictors were identified (Supplementary Table S5).

### Sensitivity analyses

The association between CGM use and ln (total PA + 1) remained significant after additional adjustment for smoking, BMI ≥ 30 kg/m², sitting time, and both acute and chronic diabetes-related complications (B = 0.45, *p* = 0.04) (Supplementary Table S2).

Similarly, CGM use remained independently associated with achieving ≥ 600 MET-min/week after adjustment for complication status (adjusted OR 2.2, 95% CI 1.2–4.5, *p* = 0.035) (Supplementary Table S3).

These findings were consistent across both absolute activity volume (minutes/week) and standardized IPAQ classification (MET-min/week).

## Discussion

### Principal findings

In this nationwide cross-sectional study of adults with type 1 diabetes (T1D) in Lithuania, CGM use was independently associated with higher physical activity levels. CGM users reported greater total physical activity, were more likely to engage in any moderate-to-vigorous physical activity (MVPA), and more frequently achieved ≥ 600 MET-min/week. Among CGM users, perceived facilitation of physical activity was strongly associated with trust in CGM accuracy during exercise and inversely associated with emotional diabetes-related distress.

Notably, only 12.7% of CGM users reported increased physical activity after CGM initiation. This observation may indicate that the observed association between CGM use and physical activity does not primarily reflect a direct behavioral change following CGM adoption, but rather differences in perceived exercise-related confidence and uncertainty among users.

### CGM and physical activity

Randomized controlled trials have consistently demonstrated that CGM improves glycemic control and reduces hypoglycemia exposure [9–11]. However, its impact on health behaviors such as physical activity remains less well established. A recent systematic review reported limited changes in overall activity volume following CGM initiation [12], whereas observational studies have suggested higher levels of vigorous activity among CGM users in certain populations [13, 14].

Our findings align with observational data, demonstrating independent associations between CGM use and both continuous and categorical activity outcomes in a real-world national cohort. These associations persisted after adjustment for demographic and clinical covariates and in sensitivity analyses. Effect sizes remained consistent across alternative model specifications, supporting the robustness of the findings. Importantly, the association between CGM use and physical activity remained stable after additional adjustment for both acute and chronic diabetes-related complications, suggesting that differences in complication burden did not explain the observed associations. Insulin pump therapy was substantially more common among CGM users and may represent a marker of greater engagement with diabetes technology and self-management. Although pump therapy was excluded from multivariable models because of strong collinearity with CGM use, some residual influence of pump use on the observed associations cannot be excluded.

Importantly, causality cannot be inferred. It is possible that individuals who are already more physically active may be more inclined to adopt advanced glucose monitoring technologies. The cross-sectional design does not allow temporal sequencing between CGM adoption and physical activity behavior.

An additional source of residual confounding may be technology adopter bias. Individuals who choose to adopt CGM may differ from non-users in socioeconomic status, health literacy, digital health engagement, treatment adherence, access to specialist diabetes care, motivation for self-management, and occupational activity patterns. Such factors may contribute both to CGM uptake and to higher physical activity levels.

### Psychological determinants and technology-related trust

Fear of hypoglycemia and glycemic variability are well-recognized barriers to exercise in T1D [4–6, 19]. Aerobic exercise is particularly associated with glucose declines [7, 8], and perceived exercise-related risk often persists despite technological advances [12]. Expert recommendations emphasize that CGM can improve safety and decision-making during physical activity [7, 20], yet behavioral engagement may depend on users’ subjective trust in device accuracy.

In our study, trust in CGM accuracy during physical activity was the strongest correlate of perceived facilitation. This supports the concept that technology-related trust may act as a psychological mediator linking device use to behavioral engagement, beyond the physiological capabilities of the technology itself.

Emotional diabetes-related distress was independently associated with lower odds of perceiving CGM as facilitating physical activity. Diabetes distress has previously been linked to impaired self-management behaviors [15], and emotional burden may influence willingness to engage in activities perceived as risky or demanding. In contrast, total diabetes distress, hypoglycemia-related confidence [16], and general psychological well-being [17] were not independently associated in adjusted models, suggesting that affective components of diabetes burden may be particularly relevant in this context.

Collectively, these findings support a model in which technological access and psychological factors jointly shape exercise-related perceptions in adults with T1D.

From a clinical perspective, these findings suggest that optimizing user confidence and addressing emotional diabetes-related distress may improve patients’ perceptions of using CGM during physical activity and may support physical activity counseling in routine diabetes care.

### Strengths and limitations

Strengths include a nationwide real-world cohort examined following national CGM reimbursement, consistent findings across multiple physical activity operationalizations, and adjustment for relevant demographic and clinical covariates.

Several limitations should be acknowledged. The cross-sectional design precludes causal inference, and reverse causation cannot be excluded. Physical activity was assessed using the self-reported IPAQ-SF, which may be subject to recall bias despite application of standard truncation procedures [18]. Reporting bias may also have differed between CGM users and non-users, as CGM users may be more engaged in self-management and health monitoring behaviors, potentially influencing the reporting of physical activity levels. Diabetes type was self-reported and not clinically verified, which may introduce potential misclassification. Insulin pump therapy was excluded due to collinearity with CGM use, and residual confounding cannot be excluded. Additionally, online recruitment through patient organizations and social media may have preferentially attracted more digitally engaged and health-conscious individuals. Because digital engagement may be associated with both CGM use and physical activity behavior, selection bias cannot be excluded. The inverse association between higher education and physical activity observed in adjusted analyses was unexpected. One possible explanation is that participants with higher educational attainment may be more likely to have sedentary occupations involving prolonged sitting time, which may not be fully captured by variables included in the models. Residual confounding and unmeasured occupational activity patterns may also have contributed to this finding. This finding should therefore be interpreted cautiously and requires confirmation in future studies. The measures used to assess perceived facilitation of physical activity and trust in CGM accuracy were study-specific and have not undergone formal psychometric validation.

## Conclusions

In this nationwide cross-sectional study of adults with T1D, CGM use was independently associated with higher physical activity levels. Among CGM users, perceived facilitation of physical activity was strongly related to trust in CGM accuracy and inversely associated with emotional diabetes-related distress. These findings highlight the potential importance of psychological and perceptual factors when interpreting associations between diabetes technology use and physical activity. Prospective longitudinal studies incorporating objective physical activity measures are needed to clarify causal pathways and underlying mechanisms.

## Supplementary Information

Below is the link to the electronic supplementary material.

Supplementary Material 1.

## Data Availability

The datasets generated and/or analyzed during the current study are not publicly available due to privacy and ethical restrictions but are available from the corresponding author on reasonable request.

## Abbreviations

CGM: Continuous glucose monitoring
DDS: Diabetes Distress Scale
FGM: Flash glucose monitoring
HbA1c: Glycated hemoglobin
IPAQ: SF–International Physical Activity Questionnaire–Short Form
MET: Metabolic equivalent
MVPA: Moderate–to–vigorous physical activity
SMBG: Self–monitoring of blood glucose
T1D: Type 1 diabetes
WHO: 5–World Health Organization–5 Well–Being Index
