# Supplementary material for: Continuous glucose monitoring use is associated with higher physical activity in adults with type 1 diabetes: a nationwide cross-sectional study
Source: Diabetol Metab Syndr. 2026 Jul 10;18:232. doi: 10.1186/s13098-026-02238-5 (PMC13729340; doi:10.1186/s13098-026-02238-5)
Supplement: Supplementary file 1 [file 13098_2026_2238_MOESM1_ESM.docx]

**SUPPLEMENTARY MATERIAL**

**Supplementary Table S1. Diabetes-related complications by CGM use (n=368)**

| **Variable** | **Total sample (n=368)** | **CGM users (n=197)** | **Non-CGM users (n=171)** | **p-value** |
| --- | --- | --- | --- | --- |
| Any chronic complication, n (%) | 186 (50.5) | 90 (47.4) | 96 (56.1) | 0.034 |
| Diabetic retinopathy, n (%) | 110 (29.9) | 43 (21.8) | 67 (39.2) | 0.001 |
| Diabetic nephropathy, n (%) | 69 (18.8) | 16 (8.1) | 53 (31.9) | 0.001 |
| Diabetic neuropathy, n (%) | 149 (40.5) | 80 (40.6) | 69 (40.4) | 0.90 |
| Any acute complication (past 6 months), n (%) | 66 (17.9) | 14 (7.1) | 52 (30.4) | <0.001 |
| Severe hypoglycemia (past 6 months), n (%) | 58 (15.8) | 11 (5.6) | 47 (27.5) | <0.001 |
| Diabetic ketoacidosis (past 6 months), n (%) | 13 (3.5) | 5 (2.5) | 8 (4.7) | 0.26 |

**Footnote:** Data are presented as n (%). Group comparisons were performed using the chi-square test. Severe hypoglycemia was defined as an episode requiring assistance from another person.

**Supplementary Table S2.** Sensitivity analysis: linear regression model for ln(total PA + 1) additionally adjusted for smoking, BMI ≥30 kg/m², sitting time, and diabetes-related complications.

| **Variable** | **B** | **SE** | **Standardized β** | **p-value** |
| --- | --- | --- | --- | --- |
| CGM use (yes vs no) | 0.45 | 0.22 | 0.13 | 0.04 |
| Age (per year) | −0.02 | 0.009 | −0.19 | 0.02 |
| Female sex | 0.29 | 0.22 | 0.08 | 0.19 |
| Education (3 levels)* | −0.11 | 0.13 | −0.06 | 0.39 |
| Employment (employed/student vs other) | −0.053 | 0.29 | −0.01 | 0.80 |
| Residence (city vs non-city) | −0.48 | 0.29 | −0.10 | 0.09 |
| Living alone (yes vs no) | 0.027 | 0.28 | 0.01 | 0.90 |
| HbA1c (%) | −0.034 | 0.036 | −0.06 | 0.30 |
| Diabetes duration (per year) | 0.005 | 0.012 | 0.03 | 0.69 |
| Any acute complication (yes vs no) | 0.06 | 0.28 | 0.013 | 0.20 |
| Any chronic complication (yes vs no) | 0.21 | 0.22 | 0.06 | 0.90 |
| Smoking (yes vs no) | 0.47 | 0.27 | 0.11 | 0.08 |
| BMI ≥30 kg/m² (yes vs no) | −0.80 | 0.39 | −0.14 | 0.03 |
| Sitting time (per hour/day) | −0.08 | 0.03 | −0.16 | 0.02 |

**Footnote:** Linear regression model with ln(total PA + 1) as the dependent variable. Model fit: R² = 0.18; adjusted R² = 0.14. VIF <2 for all variables.

**Supplementary Table S3.** Sensitivity analysis: logistic regression model for achieving ≥600 MET-min/week

| **Variable** | **Adjusted OR** | **95% CI** | **p-value** |
| --- | --- | --- | --- |
| CGM use (yes vs no) | 2.2 | 1.2–4.5 | 0.035 |
| Age (per year) | 0.93 | 0.90–0.96 | 0.001 |
| Female sex | 2.1 | 1.1–4.2 | 0.031 |
| Education (3 levels)* | 0.63 | 0.4–0.9 | 0.04 |
| Employment (employed/student vs other) | 1.2 | 0.5–3.0 | 0.6 |
| Residence (city vs non-city) | 0.4 | 0.1–1.5 | 0.07 |
| Living alone (yes vs no) | 0.5 | 0.2–1.3 | 0.16 |
| HbA1c (%) | 0.99 | 0.87–1.1 | 0.9 |
| Diabetes duration (per year) | 1.0 | 0.96–1.04 | 0.7 |
| Any acute complication (yes vs no) | 0.9 | 0.4–2.2 | 0.9 |
| Any chronic complication (yes vs no) | 1.2 | 0.6–2.2 | 0.54 |

**Footnote:** MET-minutes/week were calculated according to the IPAQ scoring protocol.

**Supplementary Table S4.** Alternative psychosocial model for perceived facilitation of physical activity among CGM users

| **Variable** | **Adjusted OR** | **95% CI** | **p-value** |
| --- | --- | --- | --- |
| Trust in CGM accuracy during physical activity (yes vs no) | 10.10 | 2.0–25.0 | 0.01 |
| Total diabetes distress (per 1-point increase) | 0.9 | 0.51–1.05 | 0.8 |
| Hypoglycemia confidence (per 1-point increase) | 2.5 | 0.97–6.0 | 0.059 |
| WHO-5 well-being (per 10-point increase) | 0.89 | 0.68–1.7 | 0.1 |
| Diabetes duration (per year) | 0.98 | 0.92–1.01 | 0.67 |
| CGM duration (increase per 12 months) | 0.79 | 0.56–1.1 | 0.2 |
| Age (per year) | 1.03 | 0.97–1.09 | 0.2 |
| Female sex | 0.90 | 0.34–2.6 | 0.86 |
| HbA1c (%) | 0.85 | 0.6–1.03 | 0.4 |

**Footnote:** Exploratory multivariable logistic regression including psychosocial variables simultaneously. Model calibration was assessed using the Hosmer–Lemeshow test. Because the number of participants in one outcome category was limited, the events-per-variable (EPV) ratio was low; therefore, estimates should be interpreted cautiously. WHO-5 was modeled per 10-point increase.

**Supplementary Table S5.** Exploratory factors associated with self-reported increase in physical activity after CGM initiation among CGM users (n=197)

| **Variable** | **OR** | **95% CI** | **p-value** |
| --- | --- | --- | --- |
| Age, years | 1.02 | 0.98–1.05 | 0.27 |
| Female sex | 1.9 | 0.77–4.89 | 0.16 |
| Diabetes duration, years | 1.05 | 1.01–1.10 | 0.01 |
| HbA1c (%) | 0.67 | 0.4–1.1 | 0.15 |
| CGM duration (per 12 months) | 1.01 | 0.98–1.35 | 0.60 |
| Emotional diabetes-related distress (per 1-point increase) | 1.04 | 0.73–1.46 | 0.80 |
| Diabetes-related distress (per 1-point increase) | 1.06 | 0.69–1.63 | 0.79 |
| Hypoglycemia-related confidence (per 1-point increase) | 1.1 | 0.60–2.20 | 0.78 |
| WHO-5 well-being (per 10-point increase) | 1.2 | 0.96–1.54 | 0.10 |
| Trust in CGM accuracy during PA (yes vs no) | 2.8 | 0.40–21.0 | 0.30 |
| Sitting time (hours/day) | 1.1 | 0.99–1.25 | 0.08 |

**Footnote:** Univariable logistic regression analyses were performed due to the limited number of participants reporting increased physical activity (n=25). Multivariable modeling was not performed.
